# A randomized trial of conventional fraction versus hypofraction radiotherapy for bone metastases from hepatocellular carcinoma

**DOI:** 10.7150/jca.28674

**Published:** 2019-07-08

**Authors:** Jian He, Shiming Shi, Luxi Ye, Guifen Ma, Xiangou Pan, Yan Huang, Zhaochong Zeng

**Affiliations:** Department of Radiation Oncology, Zhongshan Hospital, Fudan University, Shanghai, 200032, China

**Keywords:** hepatocellular carcinoma, external beam radiotherapy, conventional fractionation, hypofractionated, toxicity

## Abstract

External beam radiotherapy (EBRT) has been reported to be effective in palliating painful bone metastases, but the optimal fractions and doses for treating bone metastases from hepatocelluar carcinoma (HCC) are not established. This study aimed to compare toxicity and efficacy for conventional fraction versus hypofraction schedules. From January 2009 through December 2014, 183 patients with HCC bone metastases were randomly assigned to conventional fraction EBRT (Group A) or hypofraction radiotherapy (Group B). Study outcomes were pain relief, response rate and duration, overall survival, and toxicity incidence. Median follow-up time was 9.3 months. Response times were 6.7 ± 3.3 fractions in Group A and 4.1 ± 1.2 fractions in Group B (p <0.001). Pain relief rates were 96.7% and 91.2% in Group A and B, respectively (p=0.116). Time to treatment failure for Group A was significantly longer than Group B (p=0.025). Median overall survival was similar between two groups (p=0.628). Toxicity incidence in both groups was minimal, with no significant differences observed.

In conclusion, hypofractionated radiotherapy is safe for patients with HCC bone metastases and may achieve earlier pain relief compared to conventional radiotherapy. This protocol should be considered for patients with shorter predicted survival times.

## Introduction

An estimated 782,500 new primary liver cancer cases and 745,500 deaths occurred worldwide in 2012, with China alone accounting for about 50% of the total number of cases and deaths.^1^ Worldwide, 70-90% of primary liver cancers are hepatocellular carcinomas (HCC). The incidence of bone metastases (BMs) in patients with HCC has increased in recent years, with BMs reported in approximately 20% of patients with HCC 2,3, which may result from advances in primary tumor treatments 4.

HCC BMs are common causes of pain and other significant symptoms that diminish quality of life. Radiation therapy has been reported to provide significant pain relief from symptomatic BMs. In a previous study conducted by our group, 205 patients with HCC BMs received external beam radiotherapy (EBRT), with a complete response observed in 61 (29.8%) patients 5. Yet the most effective fractionated dose schedule for patients with HCC BMs remains unclear. The Radiation Therapy Oncology Group (RTOG) has previously studied various treatment fractionation regimens for palliation of bone metastases 6,7, and shorter treatments were found to be as effective as longer treatments for achieving pain relief. However, most of patients in these trials were suffering from prostate or breast cancer, and patients treated with a single-dose regimen of 8 Gray (Gy) were reported to have high rates of retreatment. These findings have been confirmed by other studies and meta-analysis 8-10.

To the best of our knowledge, there are few reports available which focus on palliative radiotherapy (RT) for BMs in HCC. Therefore, it is of clinical significance to determine the optimal radiation dosimetry and fractionation regimens for patients in this category. In this prospective randomized controlled study, we compared clinical outcomes for patients with HCC BMs treated with conventional versus hypofractionated radiotherapy. The aims of this investigation were to compare toxicity and efficacy between the conventional fractionation and hypofractionated schedules.

## Methods

### Study Design

This was a single-center randomized controlled trial comparing conventional fraction radiotherapy with hypofraction radiotherapy. Ethical approval for the study protocol was obtained from the research ethics committee of our hospital. This randomized clinical trial has been registered in a public database. Written informed consent was obtained from each patient prior to enrollment. Study participants were assigned using a computerized randomization table to receive either conventional fraction (Group A) or hypofraction (Group B) radiotherapy. The primary study endpoints were treatment efficacy, toxicity, and side effects. A secondary endpoint was the indication for different kinds of radiotherapy.

### Eligibility Criteria

Subjects were eligible to participate if they were between 18-85 years old; had a clinical or pathologically confirmed diagnosis of HCC with no other coinciding malignancy; had evidence of bone metastases on CT, magnetic resonance imaging (MRI), or bone scan; and had no prior history of treatment with EBRT for bone metastases. Detailed eligibility and exclusion criteria are presented in Table 1. All subjects had to be able to provide written informed consent for participation and be able to complete pain assessments.

### Treatment

Each radiation dose was administered using the ONCOR Avant-Garde Linear Accelerator (Siemens Medical Solutions, Inc. Oncology Care Systems Group). Patients were treated with linear accelerator beam energies ranging from 6-15 megavolts (MV). Dose fractionation schedules were as follows: Group A subjects received either 40 Gy in 20 fractions or 60Gy in 30 fractions, for patients without or with soft tissue formation, respectively. Group B subjects received either 28 Gy in seven fractions or 40 Gy in 10 fractions, for patients without or with soft tissue formation, respectively. All patients were treated with daily fractions on consecutive weekdays, with 5 fractions administered per week. The spine was the most frequent site of bone metastases from HCC and the normal tissue dose-volume constraints are listed as follows: spinal cord < 44 Gy; lung V20 < 20%, V5 < 60%; kidney V20 < 20%; stomach V40 < 30%; mean liver doses < 20 Gy; small intestine V40 < 1 cc; caput femoris V20 < 20% (Vx=% of the whole organ at risk receiving ≥ xx Gy). Transarterial chemoembolization (TACE) is the mainstay of treatment conducted for the tumor in the liver.

Medical examinations, pain score assessments, routine blood count, coagulation function, and blood biochemistry tests; chest/abdominal CT, MRI, B-mode ultrasound, and single-photon emission computed tomography (SPECT) were performed and analyzed for all patients throughout treatment. Positron emission tomography-CT (PET-CT) was optional for some patients.

### Simulation and Delineation

All patients underwent treatment simulation. Gross tumor volume (GTV) and plan target volume (PTV) were determined by CT, MRI, or PET-CT. Radiation fields were determined depending on each patient's metastatic condition. Helical intensity-modulated radiation therapy (IMRT) was adopted for patients with multiple metastases and for patients with metastases adjoining the spinal cord or other distant organs. For these patients, MV-class image-guided skills were used before treatment to account for set-up errors.

### Follow-up and Assessment

After treatment, patients were followed up by telephone interviews or at out-patient clinic visits once per week for the first month; once per month for the following two months; and then every six months thereafter. Pain was assessed using a numerical rating scale (NRS), according to the response categories proposed by Chow 11. Patients were asked to rate the intensity of their pain from 0 (no pain) to 10 (the worst pain imaginable). A complete response (CR) was defined as an NRS pain score of 0 at the treated site with no concomitant increase in analgesic intake. A partial response (PR) was defined as a ≥ 2-point reduction in the pain score at the treated site without an increase in analgesic intake, or with a ≥ 25% reduction in analgesic intake without increased pain. Pain progression was defined as a ≥ 2-point increase in the pain score at the treated site without a reduction in analgesic intake, or as a ≥ 25% increase in analgesic intake without a concomitant decrease in pain. An indeterminate response was defined as a response which did not reflect the definitions of CR, PR, or pain progression. Best response was defined as the best pain relief during or after the treatment course and overall response was defined as CR+PR.

### Statistical Analysis

Sample size was calculated on the basis on the following assumptions: (1) One-sided alpha = 0.05; (2) Power = 80%; (3) Anticipated response rate = 92%; (4) Non-inferiority margin = 11%; (5) Lost to follow up = 10%; (6) Randomization: 1:1 (A: B). A total of 166 patients are required. Considering 10% of lost to follow-up, the number of patients needed to provide 80% power to reject the null hypothesis that the hypofraction arm is inferior to conventional fraction arm in favor of the alternative hypothesis that the hypofraction arm is non-inferior to the conventional fraction arm are 83 and 83 for hypofraction arm and conventional fraction arm, respectively. Data are presented as the mean ± standard deviation. Patient characteristics were examined using the χ^2^ test or Fisher exact test. The Kaplan-Meier method with log-rank test was used to compare survival and TTF curves. The Cox proportional hazards regression model was applied to perform univariate and multivariate analyses. All statistical analyses were performed using the Statistical Package for Social Sciences version 19.0 (SPSS Inc, Chicago, IL). A two-sided p-value of less than 0.05 was considered statistically significant in all tests.

## Results

### Patient Characteristics

183 patients were enrolled in the study between January 2009 and December 2014. The pre-treatment characteristics of all patients are summarized in Table 2. Patient and tumor characteristics were well balanced between the two treatment arms.

### Pain Response

Table 3 shows the best response to RT for all patients by group. No statistically significant differences were found between the two treatment groups with regard to best response or overall response rate. The response time for Group B (4.1 ± 1.2 fractions) was significantly less than Group A (6.7 ± 3.3 fractions) (p <0.001).

### Time to Treatment Failure (TTF)

**Figure 1:**
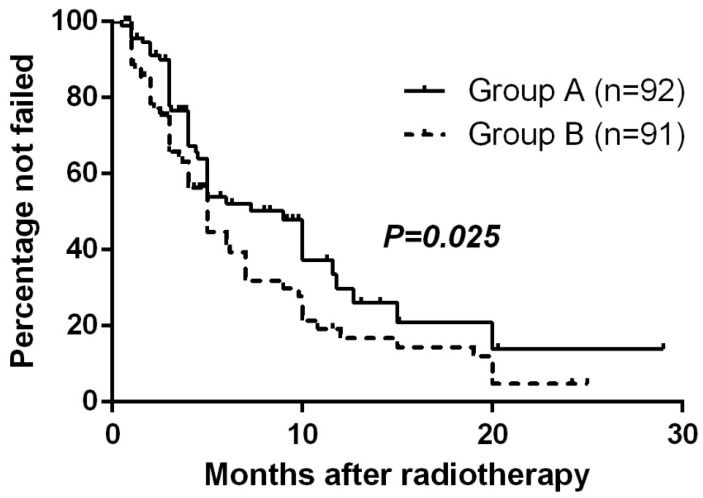
Time to treatment failure by randomized arm

At the close-out date, six patients were lost to follow-up and 106 had had treatment failure (17 remained alive and 89 had died). The estimated median TTF (95% CI) for all randomized patients was 5.0 months (3.9-6.1 mo) and the estimated percentage without failure at 1 year was 23.3%. The Kaplan-Meier method with log-rank test suggested TTF for Group A was significantly longer than Group B (p=0.025, Fig. 1).

### Toxicity

Acute radiotherapy toxicities were observed in both groups. The incidence rates for grade 0, 1, and 2 acute gastrointestinal tract toxicities in Group A were 89.1% (82/92), 7.6% (7/92), and 3.3% (3/92), respectively. In Group B, these incidence rates were 83.5% (76/91), 11.0% (10/91), and 5.5% (5/91), respectively. No Grade 3 or 4 toxicities were recorded in either group. No statistically significant differences were found between the two groups (p=0.535).

Acute hematologic toxicity was mild in both groups. The incidence rates for grade 0, 1, and 2 acute leucopenia in Group A were 93.5% (86/92), 5.4% (5/92), and 1.1% (1/92), respectively. In Group B, these incidence rates were 91.2% (83/91), 6.6% (6/91), and 2.2% (2/91), respectively. No Grade 3 or 4 toxicities were recorded in either group. No statistically significant differences were found between the two groups (p=0.790). As of the end of the study follow-up period, no late toxicities had been observed.

### Survival

**Figure 2:**
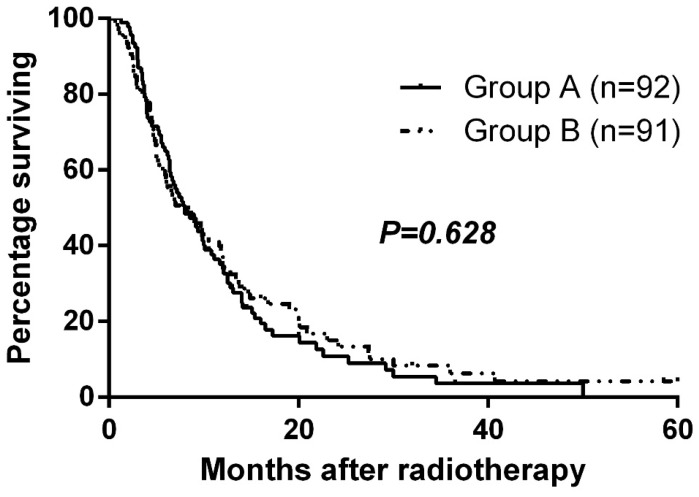
Overall survival by randomized arm

Median follow-up time was 9.3 months, and the median overall survival for the entire cohort was 8.0 months. Median overall survival times for Group A and Group B were 8.0 and 8.0 months, respectively. There was no significant difference in overall survival between the two groups (p=0.628, Fig. 2). The 1-year and 2-year survival rates were 35.1% and 10.8% for Group A and 38.7% and 15.1% for Group B. At the close-out date, six patients were lost to follow-up and the most frequent cause of death was liver failure due to recurrence or progression of local tumor.

### Prognostic Analysis

The results of univariate and multivariate Cox regression analyses of the factors related to overall survival (OS) are summarized in Table 4. Univariate analysis indicated that Karnofsky Performance Scale (KPS, p<0.001), total bilirubin (TB, p=0.028) gamma-glutamyltransferase (GGT, p=0.031), intrahepatic tumor control (p=0.001) were significant prognostic factors for OS. Multivariate analysis indicated that KPS (Karnofsky Performance Scale), TB (total bilirubin) and intrahepatic tumor control were independent prognostic factors for OS. Both univariate and multivariate analysis indicated that soft-tissue expansion and histologic type were significant prognostic factors for TTF.

## Discussion

Radiotherapy is an effective, time-efficient, well-tolerated, and cost-effective intervention that is crucial for palliative oncology care 9, yet the optimal fractionated dose scheme for patients with HCC BMs remains unclear. Different radiation schedules in common use have been proposed, namely 30 Gy in 10 fractions 12, 20 Gy in five fractions 13, and a single fraction of 8 Gy 14.

In three large, clinical randomized trials, the Radiation Therapy Oncology Group (RTOG 9714) 15, the Dutch Bone Metastasis Study 16, and the Bone Pain Trial Working Party (BPTWG) 17 have compared single fraction treatment of 8 Gy with 30 Gy in 10 fractions (RTOG 9714), 24 Gy in six fractions, and 20 Gy in five fractions (BPTWG). In the overall response rates, no differences were found between the single-irradiation arm (approximately 64% response) and the protracted radiotherapy arms (67-78% response). While these studies were published a decade ago and include various histologic types, they nonetheless still provide useful reference values. In this study, we compared outcomes following treatment with conventional fractionation versus hypofractionation. Group A received fractions of 2 Gy, while Group B received fractions of 4 Gy. In agreement with previous reports, no statistically significant differences between the two treatment schedules for best response, overall response rates, toxicity incidence, or overall survival. Conventional radiotherapy may obtain a longer duration of pain relief. However, hypofractionated radiotherapy may achieve earlier pain relief compared to conventional radiotherapy. What is more, treatment-related cost was estimated to 240 yuan per fraction, so hypofractionated radiotherapy is quite a cheap one. Given the similar observed efficacy of the two treatment schedules, these results suggest that hypofractionation might be a smarter choice for some patients with shorter predicted survival time, given its advantages in convenience and treatment-related costs. And our results suggested KPS ≤ 80, TB >17.1 umol/L and uncontrolled intrahepatic tumor might be the potential criteria for recruiting patient with shorter survival.

BMs from HCC are often characterized by soft-tissue expansion, with an abundant vascular component and elevated tumor burden 18. Nearly 40% of BMs from HCC are hypervascular soft tissue masses with adjacent destruction of the bone cortex and invasion of the surrounding muscle and/or fat tissue, changes which appear as a mixed osteolytic reaction on enhanced CT (computed tomography). 5,18,19 These soft-tissue masses are unique to HCC BMs and often cause both bone and neuropathic pain. In patients with neuropathic pain, higher RT doses are usually necessary because of the presence of soft-tissue masses 20. Our previous study suggested the retreatment rate was higher in patients with expansile soft tissue 5. Therefore, in this study, dose fractionation schedules varied between patients with and without soft tissue formation. In the conventional fractionation group, patients without soft tissue formation received a total radiation dose of 40 Gy in 20 fractions, while patients with soft tissue formation received 60 Gy in 30 fractions. In the hypofractionated group, patients without soft tissue formation received a total radiation dose of 28 Gy in 7 fractions, while patients with soft tissue formation received 40 Gy in 10 fractions.

Previous studies of radiotherapy outcomes in patients with BMs from HCC have reported that 73-99.5% of patients obtained overall pain improvement and 17-44% of patients achieved complete pain relief 17,21-23. The overall response rate of 95.9% in our study was similar to these previous reports. HCC is often complicated by liver failure, and narcotic drugs may induce hepatic coma. Therefore, radiotherapy plays a particularly important role in relieving pain from HCC BMs, by minimizing the use of narcotic drugs for pain relief.

In our study, there were no cases of severe toxicity following treatment. No statistically significant differences were found between the two groups in the incidence of acute gastrointestinal tract reactions, fatigue, or hematologic toxicity. In a randomized controlled trial conducted by the Radiation Therapy Oncology Group (RTOG 9714), the authors reported a significantly lower rate of acute toxicity after treatment with 8 Gy in one fraction compared to 30 Gy in 10 fractions; although, there was no significant difference in late toxicity (e.g., pathologic fractures) 15.

The 1-year survival rate after radiotherapy initiation or the diagnosis of BMs from HCC has been reported to be 13.8-32.4%, with a 5- to 7.4-month median survival 5,22,24-26. In patients with BMs, significant, unfavorable prognostic factors that have been reported include lower KPS 5,26, higher tumor marker levels 5, uncontrolled intrahepatic tumor 5, tumor stage 22, metastasis to other organs 22, and the presence of ascites 25. Consistent with previous reports, our results indicated that KPS, intrahepatic tumor control were significant prognostic factors impacting overall survival. GGT (gamma-glutamyltransferase) is borderline significant in multivariate analyses. These prognostic factors may be considered when determining which doses and fractions are appropriate for each patient. Performance status is a significant independent predictor of survival reported for many types of cancer, including unresectable HCC 26. GGT may induce DNA instability and subsequent oncogenesis, leading to the death of normal liver cells and loss of normal liver function 27.

Nonetheless, based on the results of this investigation, hypofractionated and conventional radiotherapy appear to provide similar treatment outcomes in patients with bone metastases from HCC, and hypofractionated radiotherapy may achieve earlier pain relief compared to conventional radiotherapy. These findings suggest that hypofractionated radiotherapy should be considered as an alternative for patients with shorter predicted survival times. Large-scale multicenter studies are warranted to substantiate and validate our results.

In conclusion, our results demonstrated hypofractionated radiotherapy is safe for patients with HCC bone metastases and may achieve earlier pain relief compared to conventional radiotherapy. This protocol should be considered for patients with shorter predicted survival times.

## Figures and Tables

**Table 1:** Eligibility Criteria

***Inclusion criteria***
1. Clinical diagnosis or pathologically confirmed hepatocellular carcinoma and no coinciding other malignancy.
2. CT, MRI, or bone scan evidence of bone metastasis at the index site.
3. Age 18-85 years old, KPS>40, without contraindication to radiotherapy.
4. Primary treatment with EBRT for bone metastases
5. Able to complete pain assessments.
6. Written informed consent.
***Exclusion criteria***
1. Pregnant or lactating women.
2. Pathological fracture.
3. Unable to complete treatment.
4. Prior chemotherapy or internal radiotherapy

Abbreviations: CT=computed tomography; MRI=magnetic resonance imaging; KPS=Karnofsky performance scale; EBRT=external beam radio therapy.

**Table 2:** Group Characteristics of the Study Patients

Clinical parameter	Group A (n=92)	Group B (n=91)	P
Age (year)			0.222
≤50	33	25	
>50	59	66	
Gender			0.112
Female	8	15	
Male	84	76	
KPS			0.588
≥80	74	76	
<80	18	15	
AFP			0.477
>400 μg/L	36	31	
≤400 μg/L	56	60	
TB			0.164
≤17.1 umol/L	84	77	
>17.1 umol/L	8	14	
Albumin			0.891
≤40 g/L	71	71	
>40 g/L	21	20	
ALT			0.601
≤40 U/L	47	50	
>40 U/L	45	41	
AST			0.500
≤40 U/L	48	52	
>40 U/L	44	39	
GGT			0.338
>60 U/L	54	47	
≤60 U/L	38	44	
ALP			0.444
≤150 U/L	17	13	
>150 U/L	75	78	
Soft-tissue expansion			0.160
No	42	51	
Yes	50	40	
Histologic type			0.257
Hepatocellular carcinoma	79	83	
No biopsy	13	8	
Intrahepatic tumor control			0.715
Uncontrolled	62	59	
Well-controlled	30	32	
Number of bone metastases			0.699
multiple	52	54	
single	40	37	
Concurrent distant metastases			0.138
Absent	54	63	
Present	38	28	

Abbreviations: KPS= Karnofsky performance scale; AFP=alpha fetoprotein; TB= total bilirubin; ALT= alanine aminotransferase; AST= aspartate transaminase; GGT=gamma-glutamyltransferase; ALP=alkaline phosphatase;

**Table 3:** Response to Radiotherapy

Parameter	Group A(n=92)	Group B(n=91)	P
Best response, n (%)			0.169
CR	41(44.6)	33(36.3)	
PR	48(52.2)	50(54.9)	
Indeterminate response	0(0)	4(4.4)	
Pain progression	3(3.3)	4(4.4)	
Overall response rate, %	96.7	91.2	0.116
Response time, fractions	6.7 ± 3.3	4.1 ± 1.2	<0.001

Abbreviations: CR= complete response; PR= partial response

**Table 4:** Univariate and Multivariate Cox Proportional Hazards Regression Analysis for Overall Survival and Time to Treatment Failure

	OS			TTF	
	HR(95%CI)	*P*		HR(95%CI)	*P*
***Univariate analysis***					
Age, years (>50 vs. ≤50)	0.886(0.633-1.241)	0.481		1.013(0.677-1.516)	0.948
Sex (male vs. female)	0.930(0.575-1.505)	0.768		1.014(0.556-1.849)	0.965
KPS (>80 vs. ≤80)	2.097(1.391-3.163)	<0.001		1.547(0.924-2.591)	0.097
AFP, μg/L (>20 vs. ≤20)	0.815(0.588-1.128)	0.217		1.264(0.844-1.892)	0.256
TB, umol/L (>17.1 vs. ≤17.1)	1.662(1.056-2.615)	0.028		1.373(0.777-2.425)	0.275
Albumin, g/L (>40 vs. ≤40)	1.240(0.862-1.784)	0.246		1.026(0.656-1.605)	0.911
ALT, U/L (>40 vs. ≤40)	1.329(0.968-1.824)	0.079		0.725(0.492-1.070)	0.106
AST, U/L (>40 vs. ≤40)	1.251(0.911-1.717)	0.166		0.907(0.615-1.336)	0.620
GGT, U/L (>60 vs. ≤60)	0.703(0.511-0.968)	0.031		0.851(0.578-1.254)	0.416
ALP, U/L (>150 vs. ≤150)	1.055(0.687-1.621)	0.807		1.014(0.593-1.731)	0.961
Soft-tissue expansion	0.889(0.648-1.220)	0.467		0.655(0.446-0.962)	0.031
Histologic type	0.840(0.492-1.435)	0.523		0.540(0.305-0.956)	0.034
Intrahepatic tumor control	0.563(0.401-0.790)	0.001		0.701(0.469-1.048)	0.083
Number of bone metastases	1.003(0.725-1.387)	0.985		1.112(0.759-1.631)	0.585
Concurrent distant metastases	1.345(0.972-1.861)	0.074		1.284(0.867-1.904)	0.212
***Multivariate analysis***					
KPS (>80 vs. ≤80)	2.105(1.392-3.183)	<0.001		NA	
TB, umol/L (>17.1 vs. ≤17.1)	1.727(1.078-2.767)	0.023		NA	
GGT, U/L (>60 vs. ≤60)	0.783(0.563-1.089)	0.146		NA	
Intrahepatic tumor control	0.553(0.394-0.778)	0.001		NA	
Soft-tissue expansion	NA			0.514(0.290-0.911)	0.023
Histologic type	NA			0.638(0.434-0.937)	0.022

Abbreviations: AFP=alpha fetoprotein; ALP=alkaline phosphatase; ALT=alanine aminotransferase; AST= aspartate transaminase; GGT=gamma-glutamyltransferase; KPS=Karnofsky performance scale; TB=total bilirubin; TTF=time to treatment failure.
